# Investigating New Sensory Methods Related to Taste Sensitivity, Preferences, and Diet of Mother-Infant Pairs and Their Relationship With Body Composition and Biomarkers: Protocol for an Explorative Study

**DOI:** 10.2196/37279

**Published:** 2022-04-27

**Authors:** Bianca Fuchs-Neuhold, Wolfgang Staubmann, Marie Peterseil, Anna Rath, Natascha Schweighofer, Anika Kronberger, Monika Riederer, Moenie van der Kleyn, Jochen Martin, Marlies Hörmann-Wallner, Irmgard Waldner, Manuela Konrad, Anna Lena Aufschnaiter, Barbara Siegmund, Andrea Berghold, Sandra Holasek, Elisabeth Pail

**Affiliations:** 1 Health Perception Lab Institute of Dietetics and Nutrition FH JOANNEUM GmbH - University of Applied Sciences Graz Austria; 2 Otto Loewi Research Center Medical University of Graz Graz Austria; 3 Institute of Midwifery FH JOANNEUM GmbH - University of Applied Sciences Graz Austria; 4 Institute of Biomedical Science FH JOANNEUM GmbH - University of Applied Sciences Graz Austria; 5 Department of Internal Medicine Division of Endocrinology and Diabetology Medical University of Graz Graz Austria; 6 Institute of Design and Communication FH JOANNEUM GmbH - University of Applied Sciences Graz Austria; 7 Institute of Analytical Chemistry and Food Chemistry Graz University of Technology Graz Austria; 8 Institute for Medical Informatics, Statistics and Documentation Medical University of Graz Graz Austria

**Keywords:** taste, preferences, nutrition, biomarkers, body composition, air displacement plethysmography, Baby Facial Actions Coding System, mother, infant, parenting, pediatrics, prenatal, postnatal

## Abstract

**Background:**

Early experiences with different flavors play an important role in infant development, including food and taste acceptance. Flavors are already perceived in utero with the development of the taste and olfactory system and are passed on to the child through breast and bottle feeding. Therefore, the first 1000 days of life are considered a critical window for infant developmental programming.

**Objective:**

The objective of our study is to investigate, both in the prenatal and postnatal period, taste sensitivity, preferences, and dietary diversity of mother-infant pairs. The explorative study design will also report on the impact of these variables on body composition (BC) and biomarkers. In contrast to conventional methods, this study involves long-term follow-up data collection from mother-infant pairs; moreover, the integration of audiovisual tools for recording infants' expressions pertaining to taste stimuli is a novelty of this study. Considering these new methodological approaches, the study aims to assess taste-related data in conjunction with BC parameters like fat-free mass or fat mass, biomarkers, and nutritional intake in infants and children.

**Methods:**

Healthy pregnant women aged between 18 and 50 years (BMI≥18.5 kg/m^2^ to ≤30 kg/m^2^; <28 weeks of gestation) were recruited from January 2014 to October 2014. The explorative design implies 2 center visits during pregnancy (24-28 weeks of gestation and 32-34 weeks of gestation) and 2 center visits after delivery (6-8 weeks postpartum and 14-16 weeks postpartum) as well as follow-up visits at 1, 3-3.5, and 6 years after delivery. Data collection encompasses anthropometric and biochemical measurements as well as BC analyses with air displacement plethysmography, taste perception assessments, and multicomponent questionnaires on demographics, feeding practices, and nutritional and lifestyle behaviors. Audiovisual data from infants’ reactions to sensory stimuli are collected and coded by trained staff using Baby Facial Action Coding and the Body Action Posture System. Birth outcomes and weight development are obtained from medical records, and additional qualitative data are gathered from 24 semistructured interviews.

**Results:**

Our cohort represents a homogenous group of healthy women with stringent exclusion criteria. A total of 54 women met the eligibility criteria, whereas 47 mother-child pairs completed data collection at 4 center visits during and after pregnancy. Follow-up phases, data analyses, and dissemination of the findings are scheduled for the end of 2023. The study was approved by the ethics committee of the Medical University of Graz (EC No 26–066 ex 13/14), and all participants provided informed consent.

**Conclusions:**

The results of this study could be useful for elucidating the connections between maternal and infant statuses regarding diet, taste, biomarkers, and prenatal and postnatal weight development. This study may also be relevant to the establishment of further diagnostic and interventional strategies targeting childhood obesity and early body fat development.

**International Registered Report Identifier (IRRID):**

DERR1-10.2196/37279

## Introduction

### Background

Many factors contribute to the development of overweight and obesity. Based on the work of Barker and Osmond in 1986 [1], recent research indicates that the risk of becoming overweight or even obese may be programmed during the prenatal and early postnatal phase. Besides genetic and hormonal factors, at the prenatal stage, the maternal environment further influences growth and can alter tissue function. To date, only limited data are available concerning prenatal and postnatal biomarkers predicting metabolic programming [2]. However, there is strong evidence that women’s prepregnancy weight and weight gain in early pregnancy are influential factors for infants’ birth weight and body composition (BC) [3,4]. Studies suggest that a healthy maternal diet and balanced nutritional status before and during pregnancy as well as physical activity have positive effects on preventing excess gestational weight gain (GWG) and a sustained impact on infants’ and adults’ health [5-7].

Therefore, the prenatal and early postnatal periods, particularly the first 1000 days from conception, are critical, wherein changes in maternal lifestyle may have far-reaching impacts [8]. The biological predispositions for sweet foods, aversion to bitter-tasting foods, and liking for salty foods in infants and children are well known [9-11]. However, taste preferences may be programmed in utero, could be modified early in life, and may play an important role in food choices later in life [11-15]. During the prenatal phase, taste buds recognizing and transmitting information to the central nervous system develop in the last trimester [16]. Maternal dietary diversity contributes to the intrauterine environment that is rich in flavors transmitted from the maternal diet to the amniotic fluid [17-19] and mother’s milk composition [20]. To identify early hedonic responses to taste stimuli in infants, studies used modified facial behavior methods, such as the Baby Facial Actions Coding System (BabyFACS), to quantify taste-elicited facial expressions in infants and their relationship to their mothers’ diet and feeding behavior [21,22].

Breastfeeding is associated with positive effects on later eating habits [23,24], adequate weight gain during infancy, and a moderately lower risk for childhood obesity [25,26]. Previous data showed that during the milk-feeding period, flavor stimulation may enhance later food acceptance. Compared to formula-fed infants, breastfed infants are more likely to accept new tastes in early childhood [27], reinforcing the effect of variety early in weaning [17,28,29]. For example, fetuses who were exposed to carrot juice for 3 consecutive weeks during the last trimester of pregnancy and during the first 2 months of lactation showed a less negative response to carrot-flavored cereals compared to plain cereals. No such preference was observed in nonexposed fetuses [17]. Thus, predispositions and preferences can be modified early through repeated exposure to flavors in amniotic fluid, mother’s milk, formula, and solid foods [30]. However, the underlying mechanisms for possible protection against later obesity by breastfeeding and the influence of early feeding practices should be further explored [31], especially to enhance the understanding of how preferences can be modified to promote a healthy diet for children [32].

Owing to the multifactorial process involved in becoming overweight or obese, sensory taste characteristics like taste preferences and sensitivity may be contributing factors. Children are reportedly predisposed to prefer food high in energy, sugar, and salt, and this contributes to the interaction between taste and fat perception, thus influencing their food intake and weight status [33,34]. The association between fat and sweet taste preferences, and weight status was determined in European children, with the odds of 50% being overweight or obese, when fat-added crackers or sugar-sweetened juices are preferred to natural crackers or natural juices by children [35-37]. There are considerable efforts focused on examining maternal lifestyle and nutritional behavior in relation to children’s and mothers’ health outcomes regarding overweight and obesity [38,39]. However, further long-term research considering various impacts and using new methodological approaches is needed to obtain taste-related data on BC, weight, and nutritional intake in infants and children [34,40].

### Objectives

The overall aim of the study is to explore new methods on taste-related dietary preferences and its association to anthropometric and biochemical parameters as well as the long-term impact on programming in utero and during early infancy. Therefore, the specific objectives are as follows:

1. To assess the prenatal data of pregnant women, including taste sensitivity and preferences, dietary intake, weight development, physical activity, and biochemical parameters; and postnatal data of mothers and their children, including taste sensitivity and preferences, dietary intake, feeding practices, weight development, BC, biochemical parameters, and lifestyle factors like physical activity, smoking, alcohol consumption, sleep, and stress perception

2. To apply new sensory methods like BabyFACS and Body Action Posture System to quantify taste-elicited expressions in infants and their relationship to their mothers’ diet and feeding behavior

3. To investigate the development of anthropometric outcomes and BC like fat-free mass (FFM) or fat mass (FM); the relationship between taste sensitivity, preferences, and dietary intake, habits, and preferences; and the association of several biochemical parameters of mothers and their children with the anthropometric outcomes, BC, taste sensitivity, and dietary preferences and habits

## Methods

### Study Design and Population

This study is an ongoing prospective longitudinal study involving 57 healthy pregnant women (≥18 years) and their offspring, with an embedded qualitative design, performed at the Health Perception Lab, a laboratory for health-relevant sensory research in Graz, Austria. The recruitment and enrollment process for low-risk pregnant women lasted from January 2014 to October 2014 in a prenatal clinic at the Styrian State Health Insurance Fund, where women underwent an oral glucose tolerance test (<28 weeks of gestation). During this visit, all pregnant women were informed in person about the study details and were given written information with contact details. To support the recruitment of participants, the study was additionally advertised on social media channels such as Facebook, on the website and in the emails of the FH JOANNEUM University of Applied Sciences and the Medical University of Graz, as well as in newspapers between January and October 2014. All documents for recruitment like posters, information folders, and advertisements were submitted for ethics approval by the review board of the Medical University of Graz.

The main inclusion criteria were pregnancy less than 28 weeks, unobtrusive oral glucose tolerance test, nonsmoking, BMI≥18.5 kg/m^2^ to ≤30 kg/m^2^, age between 18 and 50 years, and written informed consent. Detailed inclusion and exclusion criteria for the study are shown in Textbox 1.

**Inclusion and exclusion criteria of the study.**

**Inclusion criteria**

- Pregnant women 18 to 50 years of age
- Pregnancy <28 weeks from gestation
- Written informed consent
- Prepregnancy BMI≥18.5 kg/m^2^ to ≤30 kg/m^2^
- Unobtrusive oral glucose tolerance test

**Exclusion criteria**

- Birth before the 37th week or after the 42nd week of gestation
- Multiple pregnancies
- Children with severe congenital malformations or diseases
- Congenital metabolism disorders
- Drug abuse
- Drug-administered mental illnesses
- Metabolic diseases of the mother (eg, thyroid disorders)
- Autoimmune diseases of the mother (eg, Crohn disease)
- Birth complications (postpartum hemorrhage>1000 mL or eclampsia)
- Preconceptional diabetes (type 1 or 2)
- Celiac disease and wheat protein allergy of the mother
- Breast surgery and hypomasty

**Figure 1:**
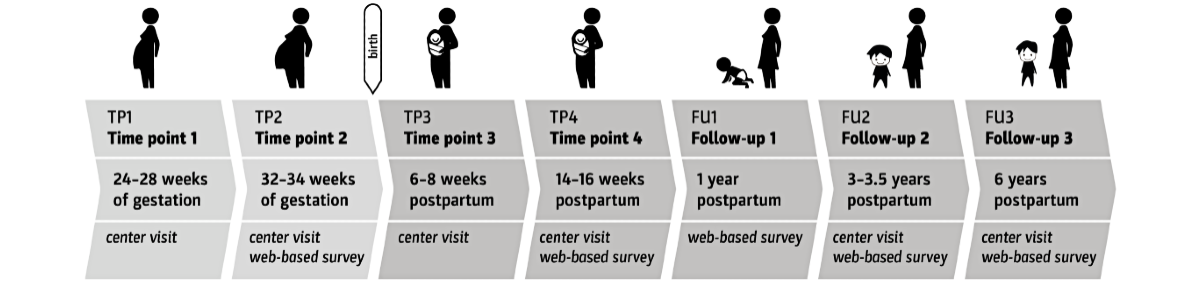
Study outline and time points of measurement.

Based on our strict inclusion and exclusion criteria, 3 of the 57 recruited women were excluded from the study. Therefore, a total of 54 women were eligible to participate in 2 center visits during their pregnancy, the first between 24 and 28 weeks of gestation (TP1) and the second in the third trimester of pregnancy, preferably between 32 and 34 weeks of gestation (TP2). Further, 2 visits were conducted with the same mother-infant pairs between 6 and 8 weeks (TP3) and between 14 and 16 weeks after delivery (TP4). The study participants are subsequently followed-up by (1) an internet-based questionnaire survey and a stool sample 1 year postpartum (FU1) and (2) a center visit to the laboratory with the mother-infant pairs at 3 to 3.5 years (FU2) and 6 years after delivery, including an extended web-based questionnaire survey (FU3), scheduled until the end of 2022. For the qualitative approach, 1 year after delivery, a purposive sampling strategy was used to record a wide variety of experiences and perspectives to obtain greater insights into women’s attitudes and beliefs regarding lifestyle changes. Within the first-year follow-up, qualitative semistructured interviews were conducted with a subsample of 24 women from the study population. Sociodemographic factors like income, educational and migration background, and age as well as further gravidity were considered and self-reported by the participants at the first center visit and updated regularly (Figure 1).

### Ethics Approval

The study protocol was approved by and registered with the ethical review board of the Medical University of Graz (EC No 26-066 ex 13/14). All participants were informed in detail about the procedures and measurements by a medical doctor and they provided written consent. Access to the generated data is restricted to the immediate research team, and only coded data stored on a secure internal server of the FH JOANNEUM University of Applied Sciences are used for analysis.

### Sample Size

We decided the number of participants for our study based on studies where sample sizes reached approximately 50-70 persons [21,40]. Additionally, feasibility reasons and recruitment experiences were considered for the laboratory setting and geographical region. Thus, we aimed to include up to 60 women.

### Data Collection

This study is conducted by an interdisciplinary team of dieticians, midwives, health scientists, information managers, nutritionists, statisticians, and biomedical analysts. Data were collected by trained health professionals twice during pregnancy, during the second and fourth month postpartum, and within the follow-up phase at 1, 3 to 3.5, and 6 years after delivery. Data collected during each stage included anthropometry, questions about health, and smoking and drug status, as well as changes regarding sociodemographic information.

### Methods of Measurement

#### Anthropometry and BC

Maternal prepregnancy BMI was calculated from the height measured with a stadiometer (seca 213, seca) without shoes, and the prepregnancy weight was obtained from the medical records or was self-reported at the first study visit to the laboratory. All the data on weight during pregnancy were obtained from the medical records (national mother-child booklet), whereas weight measurements after delivery were collected with a calibrated scale (seca 877, seca), with the participants lightly dressed and not wearing shoes. GWG was determined by subtracting the women’s prepregnancy weight and, if not available, the early first trimester weight from their last measured weight before delivery.

The infants’ length, weight, head circumference, and BC were collected in the laboratory by trained midwives. The BC, FM, FFM, and weight at TP3 and TP4 were assessed using air displacement plethysmography (ADP)(PEA POD, COSMED). Length was measured with a mobile measuring board (seca 210, seca) and head circumference with a nonflexible head circumference tape measure for infants (seca 212, seca). The BMI, fat mass index (FMI), and fat-free mass index (FFMI) were calculated in kg/m² by the system using the following equations: BMI = body mass (kg) / (body height [m])², FMI = fat mass (kg) / (body height [m])², and FFMI = fat-free mass (kg) / (body height [m])².

Follow-up data collection of the BC is ongoing for the mother-child pairs, and it is measured by ADP (BOD POD, COSMED). Additionally, triceps skinfold thickness measurements of the children are performed by trained nutritional experts using a Harpenden Skinfold Caliper in triplicate on the left arm with the arm slightly bent. In addition, the upper arm circumference of the child is determined using a tape measure. Further data on the weight, length, and head circumference at birth and beyond are being obtained from medical records.

#### Taste Perception and Facial Expression

As a measure of taste sensitivity threshold, tests for sweet and salty tastes were performed with women at TP2 and after delivery at TP3 and TP4. To keep the time burden low for participants, simplified, modified versions of the original DIN ISO 3972 and the Busch-Stockfisch version (2012) were used. The aqueous solutions were prepared according to DIN ISO 3972 using sucrose and iodine-free sodium chloride. The number of samples for the determination of taste sensitivity was modified from 10 to 5 for each stimulus, whereby the concentrations were not changed. Each sample was prepared from the respective stock solution (50 g sucrose/500 mL; 25 g sodium chloride/250 mL). Sensory tests were performed under standardized conditions in sensory booths to keep external influences as low as possible.

Preferences for the sensations of fat, sweet and fat, and salt and fat were assessed in women by making them taste crackers. Pretests aimed to find a common and well-known food item that provided the potential for experimentally modifying the fat, sugar, and salt concentrations. Considering the storage and preparation possibilities, crackers were found suitable. The crackers contained wheat flour, water, refined plant oil, salt, and sugar in specified concentrations. The basic recipe was adapted from Knof et al [41]. The participants’ preferences (sweet: sucrose-high 30% vs low 15 % and fat-15% each; salty: sodium chloride-high 2.5% vs low 1% and fat-15% each; fatty: fat-high 25% vs low 10 % and salt-0.7% each) were assessed by performing 3 pairwise comparisons of 2-alternative forced-choice tests with specified amounts of salt, sugar, and fat. Concentrations of sugar, salt, and fat content were selected according to the amounts derived from the available range of crackers in Austrian stores.

**Figure 2:**
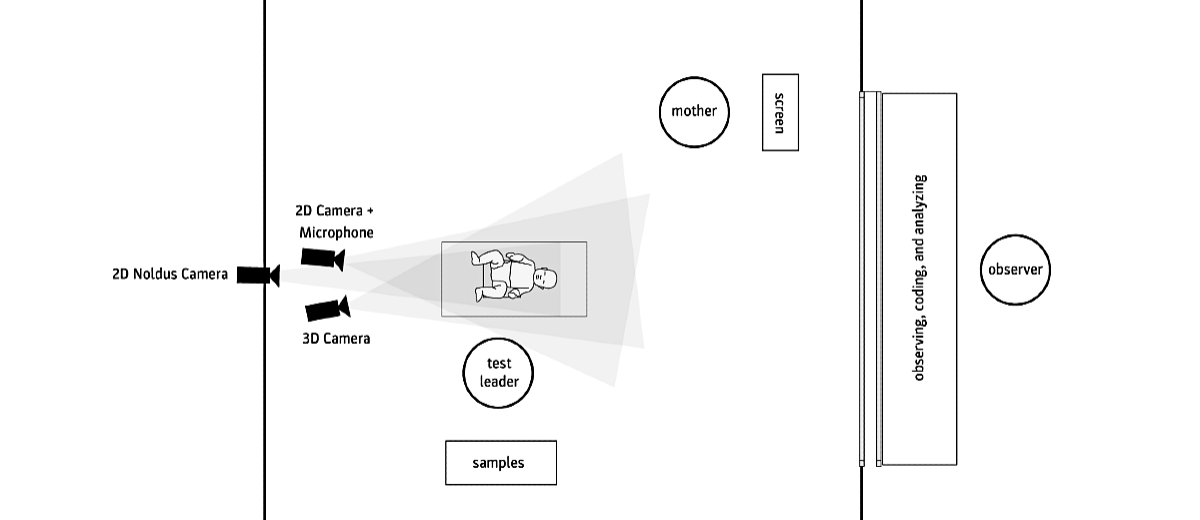
Test setting for recording infants' expressions pertaining to taste stimuli.

Infants’ taste preferences for sweet and salt were assessed at TP3 and TP4. Droplets of aqueous solutions with different concentrations (low and high) of lactose (0.2 and 0.4 mL) and sodium chloride (0.085 and 0.17 mL) were administered by a researcher with a transparent 1 mL pipette while obtaining audiovisual recordings of the infants’ reactions to the stimuli. To familiarize the infants with the test setting and method, 2 servings of water were used as the control condition. The infants’ reactions were recorded by 2 video cameras (IDS 5241VSE-C-SD32, IDS Imaging Development Systems GmbH; AXIS 211M Network Camera, Axis Communications AB), a 3D camera (Microsoft Kinect), and by a microphone (Sennheiser ME 66, Sennheiser Electronic GmbH & Co. KG). The test setting is shown schematically in Figure 2.

The infants’ reactions to the stimuli were coded by 2 trained coders using BabyFACS [21,42] for facial movements as well as the Body Action Posture System for body movements [43,44]. After the 2 coders manually coded and detailed all the distinct facial action units that the infants produced, the correspondence was checked using an intraclass correlation coefficient. Automated emotion recognition using the Noldus FaceReader 5.0 software ( Noldus Information Technology) was performed, and correlations with the coders’ observations were calculated to validate its results. In addition, the assessments of the coders and FaceReader regarding the infants' reactions to the taste stimuli were validated through additional assessment by the mothers, who were present (yet prevented from interaction) during the testing period.

At FU2 and FU3, the taste preference tests for sweet crackers were performed with the mother-child pairs, as previously described. Additionally, because of the known connection between the perception of bitter taste and the consumption of high-fat or sweet foods [45], the bitter perception of 6-n-propylthiouracil (PROP) was tested with the mother-child pairs at FU2 and FU3. Therefore, a strip of thin filter paper was impregnated with a 0.56 mM PROP solution [46] and placed on the participants’ tongue for a maximum of 20 seconds. Afterward, the subjects were asked about the perceived taste of the test strip. The so-called PROP tasters perceived the taste as negative (eg, bitter, sour, disgusting, spicy). Nontasters have no taste sensitivity to PROP [47,48].

The sensory preferences for specific food items rich in salt, sugar, and fat were additionally assessed by a recently developed and validated questionnaire called PrefQuest [49]. PrefQuest was translated into German, typical food items were adopted to the Austrian region, and it was subsequently used with permission from Deglaire et al [49]. PrefQuest quantitatively recalled the participants’ liking for the sensations of fat, fat-and-salt, and fat-and-sweet and includes four types of items: (1) liking for sweet, fatty-sweet, and fatty-salty foods, (2) preferences for the level of seasoning by adding salt, sweeteners, or fat, (3) preferences for the types of dishes on a restaurant menu, and (4) overall questions about sweet-, salt- and fat-related behaviors [49].

#### Laboratory Analyses of Biochemical Parameters

Maternal plasma and serum samples were taken at TP1 and TP2 during pregnancy and at TP3 and TP4 after delivery. Maternal breast milk was collected at TP3 and TP4. Stool samples from the mothers and children were collected at each of the center visits in the laboratory and from the children at the follow-up visits. Blood and breast milk samples were immediately stored at –20 °C until frozen and were then stored at –80 °C until analysis. Before analysis, all samples were kept at room temperature except the samples for the determination of eicosanoids, which were kept at 4 °C.

##### Eicosanoids

Blood eicosanoids were analyzed by liquid chromatography and mass spectrometry (DNA). The extraction was performed as described previously [50]. In brief, 500 μL plasma was immediately treated with 500 µL of 5% methanol/0.1% formic acid and spiked with 20 μL of internal standards (ISTDs, Cayman Europe, 95 nM). Compound extraction was performed with solid phase extraction using Oasis HLB (60 mg/30 μm, Waters). Samples were loaded onto the cartridges preconditioned with 2×1 mL methanol and equilibrated with 2×1 mL 5 % methanol/0.1 % formic acid. Each column was washed with 2×1 mL of 5% methanol/0.1% formic acid. The column was dried under vacuum and the eicosanoids were eluted with 2×0.75 mL volumes of methanol. The eluent was reduced to dryness under vacuum at 55 °C. The dried extract was subsequently reconstituted in 0.1 mL of methanol for measurement. Samples were analyzed by liquid chromatography (Agilent 1290, Agilent) coupled to electrospray ionization on a triple quadrupole mass spectrometer (Agilent 6460, Agilent). For analysis, 4 μL of the extract was injected at 5 °C. Chromatographic separation was achieved on a Waters BEH C18 column (Waters) using a flow rate of 0.4 mL/min at 40 °C during a 13-minute gradient (0-13 minutes from 25% B to 75 % B) using the solvents A, 0.1% formic acid, and B, 90:10 v/v acetonitrile/isopropanol. Electrospray ionization was performed in the negative ion mode. To detect the individual eicosanoids, dynamic multiple reaction monitoring (MRM) was performed with individually optimized MRM transitions. Data preprocessing, peak determination, and peak area integration were performed with Mass Hunter Quan (Agilent, Version B.06.00) whereas autointegration was manually inspected and corrected if necessary. The obtained peak areas of targets were corrected using appropriate ISTDs, and calculated response ratios were used throughout the analysis. Breast milk eicosanoids were determined at Lipidomix GmbH using liquid chromatography and mass spectrometry.

##### Gut Microbiota

Pea-sized human stool samples were collected in stool sample containers (containing 1 mL RNAlater solution) and stored at –20 °C. DNA was extracted using the MagNA Pure Bacterial DNA Kit (Roche) following the manufacturer’s recommendations. Next-generation sequencing (Ion Torrent 318, Thermo Fisher Scientific) and phylogenetic as well as statistical analyses were performed in the Laboratory of Diagnostic Genome Analysis at the Institute of Pathology, Medical University of Graz, Austria. In brief, next-generation sequencing was performed with Ion Torrent 318 chips. Sequencing reactions were performed on Ion Torrent PGM using the Ion 400BP Sequencing Kit (all reagents from Thermo Fisher Scientific). Sequences were split by barcode and transferred to the Torrent Suite server. Unmapped BAM files were used as inputs for bioinformatics. All sequences were initially trimmed by a sliding window quality filter with a width of 20 nucleotides and a cutoff of Q20. Reads shorter than 100 nucleotides and reads mapping to the human genome were removed using deconseq [51]. The resulting reads were subjected to error correction using the Acacia tool [52] leading to error correction of 10%-20% of the reads. Subsequently, polymerase chain reaction chimeras were removed by the usearch algorithm in de-novo and reference-based settings [53] and the final sequence files were analyzed using the QIIME 1.8 workflow script [54]. Operational taxonomic unit search was performed using the parallel_pick_open_reference_otus workflow script and the greengenes 13_8 reference database.

##### Lipid Parameters

Lipid parameters (total cholesterol, high-density lipoprotein, and triglycerides) were analyzed by enzyme immunoassay (DF27, DF48A, and DF69A, respectively) using the Siemens Dimension Xpand Clinical Chemistry Analyzer (Siemens Healthcare GmbH) according to the manufacturer’s instructions (Siemens AG). Low- density lipoprotein was calculated according to the formula of Friedewald [55].

##### Hormones

The hormones estradiol and progesterone were analyzed using an enzyme immunoassay analyzer (Abbot Architect i2000SR, Abbott GmbH) and using reagent kits 7K7225 for estradiol and 7K7725 for progesterone.

##### Adipokines and Protein

A subset of adipokines (AFABP: BioVendor, RD191036200R; Leptin: BioVendor RD191001100, Modrice; Irisin: Phoenix Ph. Inc, EK_067-52; SFRP: Cloud Clone Corp, SEC842Hu; Hepcidin: DRG Diagnostics, Hepcidin-25-HS EIA) in serum and breast milk was determined by commercially available enzyme-linked immunosorbent assays. Adipokine concentration in breast milk was either expressed per mL of breast milk or was normalized to the protein content. Measurement of the protein concentration in breast milk was performed according to the method described by Bradford [56].

##### Amino Acid Profile

Amino acid profiles were determined from maternal serum and breast milk via ion exchange chromatography followed by postcolumn derivatization with ninhydrin. The measurement was conducted at the University of Salzburg (University Clinic for Pediatrics and Adolescent Medicine) with the Biochrom 30+ Amino Acid Analyzer (Physiological System, Biochrom Ltd) according to the manufacturer’s recommendations.

#### Behavioral Variables

##### Dietary Data

Maternal diet and eating behavior during and after pregnancy were assessed via (1) the valid and reliable Inventory for Eating Behavior and Weight Problems [57] and (2) a recently developed web-based administrable food frequency questionnaire, called the Health Pregnancy Lactation-Food Frequency Questionnaire (HPL-FFQ), before the second and fourth center visits. The HPL-FFQ underwent pretests and expert validation and included the frequency (per day, per week, per month, rarely, or never) and quantity of the consumed food and beverage items during the last 3 months. For validating HPL-FFQ, further data were obtained using a 24-hour dietary recall at TP2 and TP4. The estimation of energy intake and nutritional composition of food items from HPL-FFQ and 24-hour dietary recalls was performed using the nut.s nutritional.software (dato Denkwerkzeuge, version: 1.32.30, 2015). All questionnaires were mailed 3 to 4 days prior to the women’s appointments and were checked for completeness during center visits TP2 and TP4. Additionally, the same questionnaires are mailed to the mothers in all follow-up phases.

The children’s nutritional behavior is determined by internet-based questionnaires at FU2 and FU3 using (1) the Child Eating Behavior Questionnaire [58], (2) the Food Neophobia Scale [59], and (3) a food frequency questionnaire for children (CFFQ) for the last 3 months [60]. Furthermore, for validation for the CFFQ, mothers are asked to recall all food and beverages consumed by their toddlers in the past 24 hours while visiting the laboratory at FU2.

##### Feeding Practices

Data on breastfeeding practice and duration were recorded in detail after delivery, according to the definitions of the World Health Organization [61]. Furthermore, for evaluating the exclusivity of breastfeeding, questions were asked to determine the volume of breast milk compared to other fluid intakes. Questions regarding the kind of feeding, duration and frequency of the feeds, and supplement intake, like water, tea, or solid food, were asked in 24-hour and 7-day recalls at TP3 and TP4.

Additionally, maternal feeding characteristics were assessed using the Infant Milk Feeding Questionnaire [62] at TP3 and TP4 and the Child Feeding Questionnaire [63] at FU2 and FU3.

##### Health Behavior, Physical Activity, and Media Consumption

Dlugosch and Krieger's German-language General Health Behaviour Questionnaire (FEG) [64] was used to measure further behavioral factors of postpartum women regarding alcohol, smoking, sleep, and well-being or psychosocial stress.

To determine physical activity behavior during pregnancy, questions were asked about the frequency (0 to 7 days), duration (1 to 7 hours or more), and intensity (getting out of breath and sweating; every day to never). Physical activity in mothers was assessed using the International Physical Activity Questionnaire (IPAQ), a reliable and validated questionnaire [65]. For FU1, the web-based short version was used. The instrument assesses physical activity in the last 7 days with 7 items and records the activity considering different intensity levels: (1) vigorous-intensity activities, (2) moderate-intensity activities, (3) walking, and (4) sitting. Frequency (days/week) and duration (time/day) are recorded separately for each specific activity type. The long form of the IPAQ was provided at FU2 and FU3, asking details about walking and moderate- and vigorous-intensity physical activity at four intensity levels: sitting, walking, moderate intensity (eg, leisure cycling), and vigorous intensity (eg, running or aerobics). The continuous score is expressed as the median Metabolic Equivalent of Task (MET) minutes per week: MET level × minutes of activity × events per week. Data on the children’s physical activity were collected during FU2 and FU3, according to questions from the Health Behavior in School Aged Children Questionnaire [66]. Furthermore, media consumption of children was surveyed through a question about daily use and duration reported by the mothers during FU2 and FU3.

### Stress and Coping Assessment

The Stress and Coping Inventory was designed to reliably measure the current stress, the physical and psychological consequences, and its coping. Considering the subjective postpartum stress experience, 7 items were asked, each item covering an important area of life (finance, housing, workplace/training place, partnership, family and friends, disease, and life goals). At FU2 and FU3, the items regarding coping behavior were broadened [67].

#### Semistructured Interviews

The interviews took place during the period from September 2015 to February 2016 and were conducted face to face at a convenient venue suggested by the participants. After informed consent was obtained, all interviews were recorded with a voice recorder (Philips, Voice Tracer LFH0662). The topics covered in the semistructured interview schedule explored the effects of pregnancy and childbirth on the health behavior of mothers of 1-year-old children. To represent possible changes from prepregnancy to the current life situation, including the first year as a mother, the participants surveyed were asked to provide retrospective descriptions of their health behaviors and lifestyles, focusing on nutrition and exercise.

At the end of each interview, observation memos were written up by the researcher, including subjective impressions of the interview, disturbances, and other framework conditions. Additionally, frequently mentioned key topics were continuously outlined by the researcher. Subsequently, the 24 interviews were transcribed verbatim, following predefined transcription rules. An overview of the major components of the study such as time points, data, and instruments is presented in Table 1.

**Table 1:** Major components and instruments of the study.

Time point (TP)		TP1^a^	TP2^b^	TP3^c^	TP4^d^	FU1^e^	FU2^f^	FU3^g^
**Data: instruments**								
	Screening for eligibility criteria and obtaining informed consent	✓	✓	✓	✓			
	Demographics: structured questionnaire	✓				✓	✓	✓
	Maternal health status and smoking: self-reported	✓	✓	✓	✓	✓	✓	✓
	Maternal medical history: structured questionnaire	✓						
	Maternal weight and height: medical records, weight scale, stadiometer, and self-reported	✓	✓	✓	✓	✓	✓	✓
	Paternal weight and height: reported by mother	✓						
	Pregnancy history: medical records and self-reported	✓	✓	✓	✓			
	Medication treatment of the mother and child: reported by mother	✓	✓	✓	✓	✓	✓	✓
	Birth history: medical records			✓				
	Feeding practices: structured questionnaire, IMFQ^h^, and CFQ^i^			✓	✓	✓	✓	✓
	Infant’s 24-hour and 7-day dietary intake: food recall reported by mother			✓	✓			
	Maternal nutritional behavior: HPL-FFQ^j^, IEG^k^, and PrefQuest		✓		✓	✓	✓	✓
	Maternal 24-hour dietary intake: food recall		✓		✓			
	Nutritional behavior of child: CEBQ^l^, FNS^m^, and CFFQ^n^ answered by mother						✓	✓
	Anthropometric data of child: medical records, caliper, and reported by mother			✓	✓	✓	✓	✓
	Body fat composition of child: ADP^o^			✓	✓		✓	✓
	Body fat composition of mother: ADP						✓	✓
	Blood and stool of mother: laboratory analyses	✓	✓	✓	✓		✓	
	Breastmilk: laboratory analyses			✓	✓			
	Blood and stool of child: laboratory analyses			✓	✓	✓	✓	✓
	Urine of mother and child: laboratory analyses						✓	✓
	Sensory taste perception of mother: threshold, preference, and sensitivity tests		✓	✓	✓		✓	✓
	Sensory taste perception of child: threshold, preference, sensitivity tests, and video recording			✓	✓		✓	✓
	Health behavior and stress perception of mother: FEG^p^ and SCI^q^					✓	✓	✓
	Maternal physical activity: IPAQ^r^ and structured questionnaire		✓		✓	✓	✓	✓
	Physical activity and media consumption of child: structured questionnaire answered by mother						✓	✓
	Nutritional behavior: qualitative interview of mother					✓		

^a^TP1: first center visit between 24 and 28 weeks of gestation.

^b^TP2**:** second center visit in the third trimester of pregnancy, preferably between 32 and 34 weeks of gestation.

^c^TP3: first visit conducted with the same mother-infant pairs between 6 and 8 weeks.

^d^TP4: second visit conducted with the same mother-infant pairs 14 and 16 weeks after delivery.

^e^FU1: first follow-up involving an internet-based questionnaire survey and collection of a stool sample 1 year postpartum.

^f^FU2: a center visit to the laboratory with the mother-infant pairs at 3 to 3.5 years.

^g^FU3: visit 6 years after delivery including an extended web-based questionnaire survey.

^h^IMFQ: Infant Milk Feeding Questionnaire.

^i^CFQ: Child Feeding Questionnaire.

^j^HPL-FFQ: Health Pregnancy Lactation-Food Frequency Questionnaire.

^k^IEG: Inventory for Eating Behavior and Weight Problems.

^l^CEBQ: Child Eating Behavior Questionnaire.

^m^FNS: Food Neophobia Scale.

^n^CFFQ: food frequency questionnaire for children.

^o^ADP: air displacement plethysmography.

^p^FEG: Dlugosch and Krieger's German-language General Health Behaviour Questionnaire.

^q^SCI: Stress and Coping Inventory.

^r^IPAQ: International Physical Activity Questionnaire.

### Data Analysis and Dissemination

Quantitative data analyses will be performed using SPSS Version 27 (IBM Corporation). Baseline data will be presented descriptively. Continuous variables will be presented as means (with standard deviations). Categorical variables will be presented as absolute numbers and rates. Mean nutrition values are derived by participant-individual averaging. Parameters that are not normally distributed will be either log transformed or analyzed using nonparametric methods. Group comparisons will be performed using chi-square tests for categorical characteristics and *t* tests, ANOVA, or Mann-Whitney *U* tests for continuous variables. Correlation and regression analyses are used to investigate the association between various exposure variables of interest and the longitudinal outcomes. Furthermore, associations between the aforementioned parameters as well as the BMI of the children, the collected biomarkers, and children’s taste perception, sensitivity, and preferences were investigated by explorative data analysis. Associations with maternal weight gain and the children’s weight will be analyzed using ANOVA models adjusted for possible confounders. Relationships between the mothers' senses or preferences of taste during and after pregnancy and the children’s taste preferences are shown in contingency tables. Univariate models will be established initially to explore the association between the exposure variables and each outcome. Effects of potential confounders are adjusted in the multivariable models.

Qualitative data will be analyzed using f4analyse, a tool for data coding, sorting, and categorizing. A thematic approach and triangulation with the quantitative data (if possible) will be used to identify themes informed by the methods of Braun and Clarke [68].

Analyses on taste-related data and their association with BC, weight, and food intake in mother-infant pairs are scheduled starting with the completion of FU3 by the end of 2022. Subsequently, the dissemination of the results obtained from the newly developed methods should be started first, for example, the validation of the HPL-FFQ. Findings on taste sensory methods related to nutrition, biomarkers, and BC should be published in peer-reviewed journals and presented at high-level conferences by the end of 2023. Therefore, dissemination of results will occur regardless of the outcomes (positive or negative).

## Results

Out of the 57 recruited healthy women, 54 participated in the explorative pilot study, whereas 47 mother-child pairs completed data collection from 4 center visits. The follow-up phase is scheduled for the end of 2022. Data analysis and dissemination of the main findings should be completed by the end of 2023. This study was conducted according to the guidelines laid down in the Declaration of Helsinki, and all procedures involving human subjects were approved by the local ethics committee of the Medical University of Graz (EC No 26-066 ex 13/14). Written informed consent was obtained from all subjects. The study was funded by the Austrian Research Promotion Agency (FFG, grant 839098). The study contents have undergone peer review by the funding body and the funding sources were not involved in conducting the research and will not have any role during execution, analyses, interpretation of the data, or in the decision to publish the results.

## Discussion

### Overview

One of the many factors influencing dietary habits are sensory experiences, with the possibility of shaping and modifying flavor perception and developing strategies for promoting healthy diet in children with a positive food variety [30]. Several studies indicate that prenatal exposure and postnatal feeding practices, especially breastfeeding, have been associated with flavor stimulation and moderately lower childhood obesity [6,8]. To analyze the multifactorial process with respect to overweight or obesity, new methodological approaches that consider different influences are needed to study taste-related data and their association with BC, weight, and food intake in infants and children [34,40].

This paper focuses on the study protocol, providing details on the measurement methods. Assessing the mother’s and child’s taste perception, including audiovisual data and the BC at several time points, is an innovative approach and can provide insights into a different and scarcely explored field of health-related sensory research to prevent childhood and adult obesity development. Long-term data are collected and analyzed under standardized conditions in the prenatal period from healthy mothers, following strict inclusion and exclusion criteria, and in the postnatal period from mother-infant pairs by an interprofessional team to reveal insights for follow-up studies and provide an interdisciplinary understanding of the factors influencing food preferences and weight development in early life.

The anticipated main findings of the study should address the application of new sensory assessment methods using BabyFACS to identify taste-specific data in infants and its association with diet, BC-related biomarkers with a focus on eicosanoids, and fat-related indices like FMI and FFMI as well as the correlation of breast milk parameters with the infants’ intestinal microbiota, which are potentially involved in the early prediction of the development of childhood overweight and obesity.

### Potential Strengths and Limitations

This explorative study combines long-term quantitative and qualitative data collection of sensory, anthropometric, nutritional, and biochemical parameters of mother-infant pairs and is characterized by several strengths. First, the study population is small and homogeneous because of selection using strictly defined and comprehensive inclusion and exclusion criteria; the data are thoroughly documented with a follow-up period of 6 years. Nevertheless, the results need to be interpreted carefully, but the sample homogeneity and detailed descriptions provided by the participants are sufficient for exploratory analyses and method development. Thus, the development of an audiovisual test setting for recording infants' expressions pertaining to taste stimuli between the sixth and eighth week after birth and the processing of these data using BabyFACS and the Body Action Posture System is a special innovation in this study. In addition, BC could be measured accurately with ADP. Further, prenatal and postnatal nutrient intakes are assessed via a recently validated but not yet published instrument called the HPL-FFQ. For validation purposes, 2 24-hour recalls were performed as in-depth face-to-face interviews by a trained nutrition expert. Moreover, recall errors and bias in the dietary intake and physical activity assessments cannot be ruled out completely. Another limitation is the fact that the data categories retrieved from the medical records may be not exhaustive and may show heterogeneous quality due to different data collection procedures. Information such as the prepregnancy body weight and body weight during the first trimester could not be objectively measured because the first visit was set from 24 to 28 weeks of gestation. Furthermore, postponements of visits must be expected and may influence the week of pregnancy in the defined visit period.

### Future Directions

We assume that the identified parameters in our explorative study could be useful for elucidating connections between maternal metabolic and nutritional status and infant development. Therefore, future studies must focus on determining the relationships between the mother’s and child’s taste parameters, including audiovisual data, candidate biomarkers from maternal blood or breast milk, infants’ fat-related indices, and gut microbiota composition, especially in the first year of life. Additionally, the findings from our study could be an important step toward the establishment of further diagnostic and interventional strategies targeting childhood obesity and early body fat development. Although there are many unanswered questions related to the complex development of obesity, these results might encourage the confirmation of the identified parameters within a larger cohort to quantify the effect of early stimulation of taste and preferences as well as assess potential differences and similarities between population groups.

## Appendix

Multimedia Appendix 1 Peer-review report.

## Abbreviations

ADP: air displacement plethysmography
BabyFACS: Baby Facial Action Coding System
BC: body composition
FMCFFQ: food frequency questionnaire for children
FEG: Dlugosch and Krieger's German-language General Health Behaviour Questionnaire
FFM: fat-free mass
FFMI: fat-free mass index
FM: fat mass
FMI: fat mass index
GWG: gestational weight gain
HPL-FFQ: Health Pregnancy Lactation-Food Frequency Questionnaire
IPAQ: International Physical Activity Questionnaire
ISTD: internal standard
MET: Metabolic Equivalent of Task
MRM: multiple reaction monitoring
PROP: 6-n-Propylthiouracil
