# Supplementary material for: Investigating New Sensory Methods Related to Taste Sensitivity, Preferences, and Diet of Mother-Infant Pairs and Their Relationship With Body Composition and Biomarkers: Protocol for an Explorative Study
Source: JMIR Res Protoc. 2022 Apr 27;11(4):e37279. doi: 10.2196/37279 (PMC9096638; doi:10.2196/37279)
Supplement: Multimedia Appendix 1 [file resprot_v11i4e37279_app1.pdf]

4. Ausschreibung COIN Programmlinie Aufbau
Auszüge aus den Fachgutachten (nicht-vertraulicher Teil)
Projektnr.: 839098
Kurztitel: HealthPerceptionLab

FACHGUTACHTEN 1

Frage 1:
Ausgangslage

Stärken:
Bislang gibt es in Österreich keine Institution, die die Zusammenhänge zwischen der Entwicklung von Geschmackspräferenzen und Adipositasentstehung systematisch erforscht. Die Einrichtung des HealthPerception Lab könnte diese Lücke schließen und Anschlussfähigkeit und die internationale Forschung herstellen.
Eine weitere Stärke ist der Bogen von eher grundlegender Forschung (Schwerpunkt 1) bis zu anwendungsorientierter Umsetzung (Schwerpunkt 2 und 3). Da sich eine solche Verknüpfung sonst eher selten finden, ist auch hier ein erhebliches Innovationspotential zu vermuten.

Schwächen:
Der Stand der Forschung zur frühkindlichen bzw. pränatalen Geschmackswahrnehmung wird bestenfalls sehr rudimentär dargestellt. Daher lässt sich das Innovationspotential gegenüber dem bisherigen Kenntnisstand ohne aufwändige eigene Recherche nicht wirklich beurteilen.

Frage 2:
Wissenschaftliche Qualität

Stärken:
Die Entwicklung und Validierung von methodischen Ansätzen zur Durchführung sensorischer Untersuchungen bei Kleinkindern (3 D Babytracker) ist ein absoult herausragendes Highlight des Antrags. Aufgrund der vorhandenen Kompentenz erscheint es realistisch, dass dieser äußerst bedeutsame Innovationsschritt tatsächlich erreicht werden kann.

Es kann erwartet werden, dass diese Methodik im weiteren Verlauf tatsächlich zu wichtigen neuen Erkenntnissen führt. Bislang ist dies aber noch nicht spezifisch ausgeführt (siehe Schwächen).

Von einer Reihe von Kooperationspartnern liegen Interessenbekunden vor, die sich vor allem auf die anwendungsorientierten Aspekte (FSP 2 und 3) des Projektes beziehen.

Schwächen:
Die Darstellung des 1. Forschungsschwerpunktes wirkt sehr ambitioniert, ist aber wenig präzise. Es werden eine Fülle von verschiedenen Parametern erhoben, die mit der Gewichtsentwicklung von Kindern korreliert werden sollen. Aber es gibt keine präzisen Hypothesen oder Forschungsfragen, welche Zusammenhänge nun genau erwartet und daher überprüft werden sollen (und welche nicht). Der Ansatz "wir korrelieren alles mit allem" ist nicht überzeugend.

Weiterhin stellt sich die Frage, ob die Gewichtsentwicklung in den ersten 6 Lebensmonaten ein hinreichend guter Prädiktor für die Gewichtsentwicklung im weiteren Lebenslauf ist. Hierfür wird keine Evidenz angeboten.

Es wird eine Fallzahl von 200 Probanden im 1. FSP angestrebt. Für diese Fallzahl gibt es keine Stichprobenberechnung, sie erscheint eher willkürlich.

Der 2. Forschungsschwerpunkt hat zum Ziel gesundheitsorientierte Produkte (vor allem mit geringerem Fett- und Zuckergehalt) für (Klein-)kinder zu entwickeln. Der Antrag fokussiert hier ausschließlich auf die sensorischen Aspekte solcher Produkte. Angesichts der Gesetzmäßigkeiten von Präferenzentwicklungen (mere exposure, social influence etc.) stellt sich aber die Frage, ob das überhaupt die entscheidenden Gesichtspunkte sind, oder ob nicht Marketingaspekte bzw. -konzepte Präferenzen viel wirksamer beeinflussen. Marketingaspekte im Sinn "Meinung über Produkte" werden bislang jedoch überhaupt nicht berücksichtigt.

Frage 3:
Qualität der Planung

Stärken:
Projektplanung in Arbeitspaketen, Zeitplan und Meilensteinplan erscheint insgesamt nachvollziehbar, schlüssig und angemessen.

Die Projektbeteiligten erscheinen insgesamt hinsichtlich Kapazität und Kompetenz gut integriert.

Die Kosten für die materielle Infrastruktur sind für das Projekt sehr gut berechtigt und nachvollziehbar.

Schwächen:
Es ist nachvollziehbar, dass der Großteil der Kosten durch Personalaufwand verursacht wird. Allerdings ist der Personalaufwand insgesamt sehr hoch veranschlagt und nicht immer in dieser Höhe nachvollziehbar.

Für das AP 2 Literaturrecherche wird ein Arbeitsaufwand von ca. 2620 Std. veranschlagt (bei 1500 h pro Jahr = 1,7 Jahre Vollarbeitszeit), dieser Aufwand erscheint deutlich überhöht.

AP4 Aufbau der Infrastruktur (insg. 2020 h): hier sind eine Vielzahl von Leuten mit bis zu 200 h beteiligt. Es ist nicht klar, was jeweils der Beitrag der Betreffenden ist und wozu dieser nötig ist.

AP6 Geschmacksentwicklung: hier sind für eine Dauer von insgesamt 2 Jahren 7750 h (=5,1 Jahre Vollzeitarbeit) eingeplant. Es ist einleuchtend dass dieses AP sehr arbeitsaufwändig ist. Aber in diesem Umfang ist der Aufwand ohne weitere Erläuterung nicht nachvollziehbar.

Frage 4:
Ökonomisches Potenzial und Verwertung

Stärken:
Es wird eine Vielzahl von potentiellen Umsetzungs- und Verwendungsmöglichkeiten benannt, die durch beigefügte Interessenerklärungen gestützt und plausibel gemacht werden.
Die Einschätzung des Umsetzungspotentials erscheint realistisch

FACHGUTACHTEN 2

Frage 1:
Ausgangslage

Stärken:
Beantragtes Health-Perception-Lab ist hoch innovativ.

Schwächen:
Oberflächliche Darstellung des State-of-the-Art.

Kommentare zum Kriterium:
Der State-of-the-Art insbesondere im Bereich Sensorik- und Konsumentenverhaltensforschung ist nur oberflächlich dargestellt. Das beantragte Health-Perception-Lab allerdings beinhaltet - im deutschsprachigen Raum und spezialisiert auf den prä- und postnatalen Entwicklungszeitraum - einen sehr hohen Innovationsgehalt.

Frage 2:
Wissenschaftliche Qualität

Stärken:
Interdisziplinäre Herangehensweise

Schwächen:
Über weite Strecken oberflächliche Darstellung, Quellen-/Literaturauswahl punktuell und unklar.

Allgemeine Kommentare zum Kriterium:
Die wissenschaftliche Qualität des Antrags weist deutliche Schwächen auf. Die Auswahl der wenigen Literaturzitate erscheint zufällig und einseitig. Der wissenschaftliche Hintergrund des Antrags bleibt tw. oberflächlich.

Frage 3:
Qualität der Planung

Stärken:
interdisziplinäre und institutionelle Vernetzung, hoch innovative Babytracker-Software

Schwächen:
Vielzahl von Fragestellungen, daher tw. oberflächlich; diverse methodische Aspekte bleiben unberücksichtigt (Fallzahlschätzung 200 Probandinnen, ethisch-rechtiche Überlegungen zur sensorischen Forschung mit Kindern, ...), tw. inhaltliche Fehlaussagen (z. B. Kinder und Kleinkinder haben keine speziellen diätetischen Anforderungen), keine Aussagen zur methodischen Herangehensweise an zeitliche Entwicklungen, Phase des Literaturstudiums außerordentlich lang und teuer, ...

Allgemeine Kommentare zum Kriterium:
Die Qualität der Planung hat deutliche Schwächen. Der Projektantrag sollte sich ggf. auf die Einrichtung des hoch innovativen Health-Perception-Lab und erste Pretests beschränken. Dies würde die Umsetzbarkeit deutlich erhöhen. Die Kostenplanung wird weitgehend als realistisch eingeschätzt. Lediglich die Phase der Literaturarbeit ist deutlich zu lange und zu teuer, mit Einschränkung gilt dies auch für die Reisekosten. Die Zusage der Übernahme der Baukosten durch die FH ist aus den verfügbaren Unterlagen nicht eindeutig ersichtlich. Eine stärkere Expertise im Bereich praktischer Konsumentensensorik und Konsumentenverhaltensforschung ist zeitnah und dringend nötig.

Frage 4:
Ökonomisches Potenzial und Verwertung

Stärken:
Potenzial liegt überwiegend in der herstellernahen Grundlagenforschung.

Allgemeine Kommentare zum Kriterium:
Das Potenzial liegt hauptsächlich im Bereich Grundlagenforschung für Lebensmittelherstellung und Ernährungsbildung und -erziehung. Dies wird realistisch dargestellt.
